# Repeated Exposure to Methamphetamine, Cocaine or Morphine Induces Augmentation of Dopamine Release in Rat Mesocorticolimbic Slice Co-Cultures

**DOI:** 10.1371/journal.pone.0024865

**Published:** 2011-09-30

**Authors:** Takayuki Nakagawa, Yuichi Suzuki, Kazuki Nagayasu, Maiko Kitaichi, Hisashi Shirakawa, Shuji Kaneko

**Affiliations:** Department of Molecular Pharmacology, Graduate School of Pharmaceutical Sciences, Kyoto University, Kyoto, Japan; University of Chicago, United States of America

## Abstract

Repeated intermittent exposure to psychostimulants and morphine leads to progressive augmentation of its locomotor activating effects in rodents. Accumulating evidence suggests the critical involvement of the mesocorticolimbic dopaminergic neurons, which project from the ventral tegmental area to the nucleus accumbens and the medial prefrontal cortex, in the behavioral sensitization. Here, we examined the acute and chronic effects of psychostimulants and morphine on dopamine release in a reconstructed mesocorticolimbic system comprised of a rat triple organotypic slice co-culture of the ventral tegmental area, nucleus accumbens and medial prefrontal cortex regions. Tyrosine hydroxylase-positive cell bodies were localized in the ventral tegmental area, and their neurites projected to the nucleus accumbens and medial prefrontal cortex regions. Acute treatment with methamphetamine (0.1–1000 µM), cocaine (0.1–300 µM) or morphine (0.1–100 µM) for 30 min increased extracellular dopamine levels in a concentration-dependent manner, while 3,4-methylenedioxyamphetamine (0.1–1000 µM) had little effect. Following repeated exposure to methamphetamine (10 µM) for 30 min every day for 6 days, the dopamine release gradually increased during the 30-min treatment. The augmentation of dopamine release was maintained even after the withdrawal of methamphetamine for 7 days. Similar augmentation was observed by repeated exposure to cocaine (1–300 µM) or morphine (10 and 100 µM). Furthermore, methamphetamine-induced augmentation of dopamine release was prevented by an NMDA receptor antagonist, MK-801 (10 µM), and was not observed in double slice co-cultures that excluded the medial prefrontal cortex slice. These results suggest that repeated psychostimulant- or morphine-induced augmentation of dopamine release, *i.e.* dopaminergic sensitization, was reproduced in a rat triple organotypic slice co-cultures. In addition, the slice co-culture system revealed that the NMDA receptors and the medial prefrontal cortex play an essential role in the dopaminergic sensitization. This *in vitro* sensitization model provides a unique approach for studying mechanisms underlying behavioral sensitization to drugs of abuse.

## Introduction

Psychostimulants (e.g. amphetamines and cocaine) and opiates (e.g. morphine) share the ability to cause drug dependence and addiction. In rodents, repeated intermittent exposure to psychostimulants and morphine leads to progressive augmentation of their locomotor activating effects. This phenomenon, termed behavioral sensitization, is thought to underlie certain aspects of drug addiction [1]. It is well established that the drug-associated behaviors including locomotor and rewarding effect of such drugs depend on their ability to elevate extracellular dopamine levels in the mesocorticolimbic dopaminergic neurons that originate in the ventral tegmental area (VTA) and project to the nucleus accumbens (NAc), the medial prefrontal cortex (mPFC) and other forebrain regions [2].

The effect of amphetamines on dopamine release is mainly attributed to their binding to and reversal of dopamine transporter (DAT) function, resulting in both reuptake inhibition and release of dopamine [3], while cocaine inhibits reuptake of dopamine at the mesocorticolimbic dopaminergic nerve terminals. Opioids inhibit the inhibitory γ-amino-butyric acid (GABA) interneurons in the VTA through μ-opioid receptor activation and subsequently activate the mesocorticolimbic dopaminergic neurons [4]. A body of evidence suggests that repeated exposure to psychostimulants and morphine augments the dopamine release in the NAc [5]–[8] and mPFC [9], [10], which contributes to their behavioral sensitization [11], [12], although the altered dopamine release in the mPFC under sensitization state is dependent on the regimen and withdrawal days [10], [13]–[15]. On the other hand, the mesocorticolimbic dopaminergic neurons could be regulated by the glutamatergic neurons through *N*-methyl-d-aspartate (NMDA) and non-NMDA receptors, which are innervated from the limbic and cortical areas, such as the mPFC, hippocampus and amygdala to the NAc [16], [17]. It is suggested that neuroadaptations in interaction between the mesocorticolimbic dopaminergic and glutamatergic system in the NAc by repeated exposure to psychostimulants and opioids play an important role in drug addiction [11], [18], [19].

Many aspects of drug addiction have been assessed by *in vivo* experiments in whole animals, because these addiction-related phenomena are thought to be due to long-term alterations in psychological behavior caused by synaptic plasticity in the mesocorticolimbic dopaminergic neurons. Acute effects of drugs of addiction on dopaminergic function have been extensively assessed by *in vitro* experiments using cell lines expressing DAT [20], [21], primary cultures of dopaminergic neurons [22] or acute striatal or mesencephalic slice preparations [23]–[25]. However, *in vitro* culture systems that recapitulate cell-to-cell interactions between different parts of the brain are anticipated to provide much valuable data about the long-term effects of drugs, facilitating investigations of the neural plasticity underlying drug addiction.

To this end, Maeda et al. reconstructed the mesocorticolimbic system using rat triple organotypic slice co-cultures of the mesencephalic slice including the VTA, the ventral striatal slice including the NAc, and the mPFC slice [26]. Using an extracellular recording technique with a multi-electrode dish, they showed that the triple slice co-cultures retained a functional corticoaccumbens glutamatergic pathway from the mPFC to the NAc. Furthermore, they found that cocaine attenuated the synaptic activity of the corticoaccumbens pathway through activation of D_1_-like, but not D_2_-like, dopamine receptors [26]. These findings demonstrate that the triple slice co-cultures retain functional interactions between the mesocorticolimbic dopaminergic and corticoaccumbens glutamatergic pathways in the NAc. Thus, the VTA/NAc/mPFC triple slice co-cultures are ideal for the analysis of acute and chronic effect of drugs of abuse.

To explore the utility of the triple slice co-cultures for studying certain processes relevant to behavioral sensitization *in vitro*, we examined the effects of acute and chronic effects of psychostimulants and morphine on dopamine release. Here we report that repeated exposure of the triple slice co-cultures to methamphetamine (METH), cocaine or morphine augmented their dopamine releasing effects, *i.e.* dopaminergic sensitization. Furthermore, we examined the involvement of NMDA receptors and the mPFC in the dopaminergic sensitization.

## Results

### Histological studies

**Figure 1:**
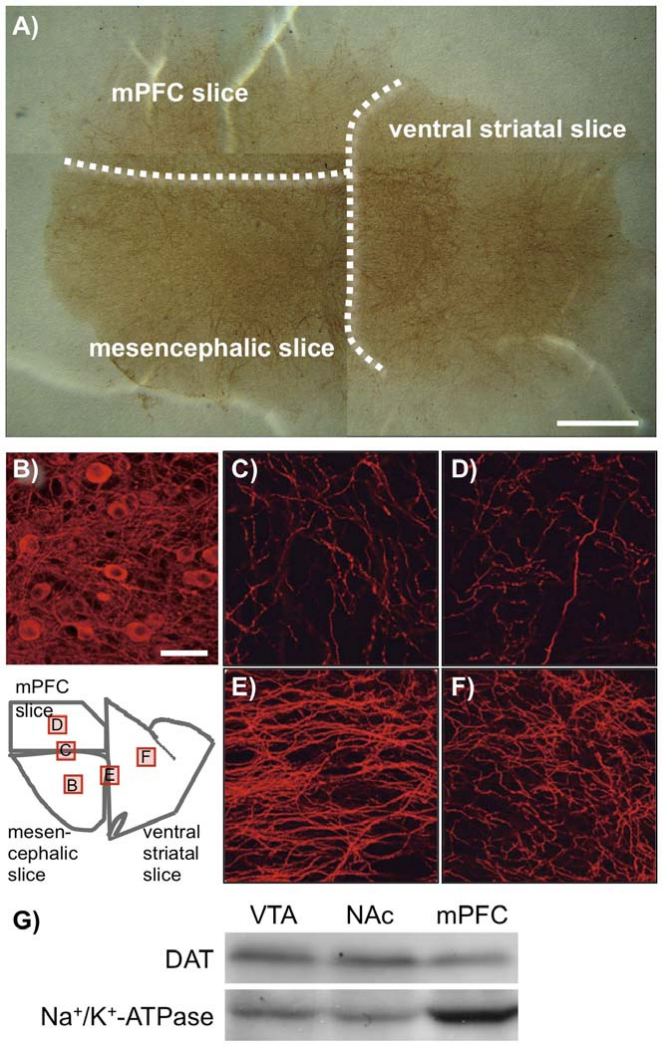
Immunohistochemistry for TH in the VTA/NAc/mPFC triple slice co-cultures. (A) Photomicrograph for TH immunoreactivity by 3,3′-diaminobenzidine staining in a representative triple slice co-culture. The dotted lines represent the borders of mesencephalic, ventral striatal and mPFC slices. Scale bar = 1 mm. (B–F) Fluorescence photomicrographs for TH immunoreactivity in the (B) VTA in the mesencephalic slice, (C) border of mesencephalic and mPFC slices, (D) mPFC slice, (E) border of mesencephalic and ventral striatal slices and (F) NAc in the ventral striatal slice. Square frames in the illustration of the VTA/NAc/mPFC triple slice co-culture indicate the regions corresponding to the photomicrographs (B)–(F). Scale bar = 50 µm. (G) The expression of DAT protein (approximately 75 kDa) in the VTA, NAc and mPFC parts separated from the triple slice co-cultures was determined by Western blotting (upper panel). Lower panel shows blots for Na^+^/K^+^-ATPase (approximately 100 kDa) as a loading control.

To assess the reconstruction of the mesocorticolimbic dopaminergic neurons in the VTA/NAc/mPFC triple slice co-cultures, immunostaining was performed for tyrosine hydroxylase (TH), the rate-limiting enzyme in the biosynthesis of catecholamines (Fig. 1A–F). TH-positive cells and neurites were clearly observed by bright field or fluorescence microscopy. A number of TH-positive cell bodies were localized in the VTA of the mesencephalic slices. The TH-positive neurites were abundantly observed in the mesencephalic slices, and they were also observed in the ventral striatal and mPFC slices, which crossed the VTA-NAc and VTA-mPFC borders. Furthermore, Western blot revealed that DAT was expressed in the VTA, NAc and mPFC parts of the triple slice co-cultures (Fig. 1G).

### Effects of single treatment with psychostimulants and morphine on dopamine release

**Figure 2:**
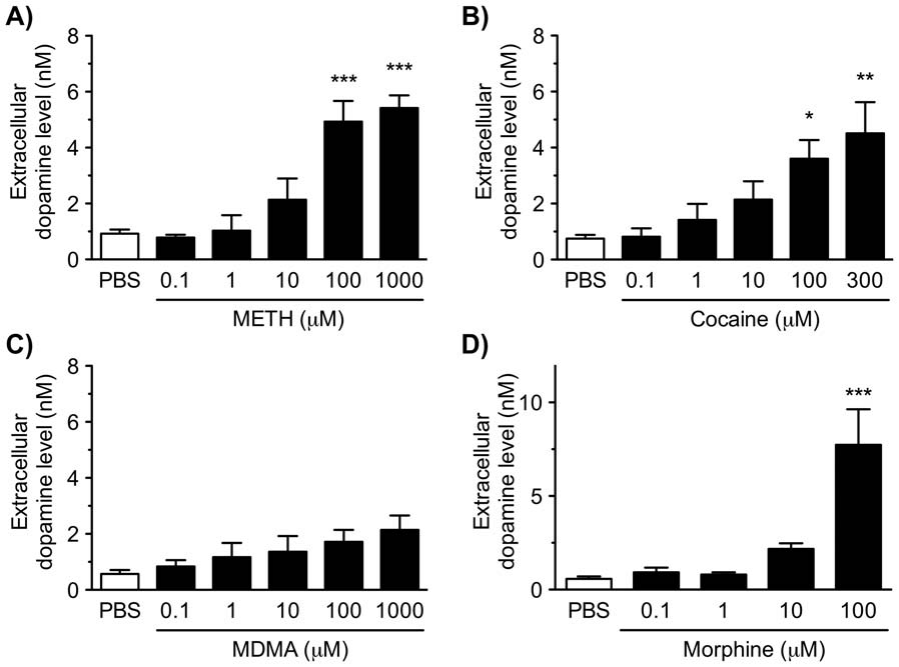
Effect of single treatment with METH, cocaine, MDMA or morphine on the extracellular dopamine level in the VTA/NAc/mPFC triple slice co-cultures. The triple slice co-cultures were treated with METH (0.1–1000 µM; A), cocaine (0.1–300 µM; B), MDMA (0.1–1000 µM; C) or morphine (0.1–1000 µM; D) for 30 min in KRH buffer, and then the extracellular dopamine levels were determined. Values represent means of the dopamine concentration ± S.E.M. METH, *n* = 3–7; cocaine, *n* = 3–10; MDMA, *n* = 3–5; morphine, *n* = 3–5. *p<0.05, **p<0.01, ***p<0.001 versus PBS-treated slice co-cultures.

The effects of single treatment of the VTA/NAc/mPFC triple slice co-cultures with METH, cocaine, MDMA or morphine on dopamine release were examined (Fig. 2). Single treatment with METH (0.1–1000 µM), cocaine (0.1–300 µM) or morphine (0.1–100 µM) for 30 min significantly increased the extracellular dopamine level in a concentration-dependent manner (METH; *F*_5,22_ = 11.22, *p*<0.001, cocaine; *F*_5,40_ = 3.857, *p*<0.01, morphine; *F*_4,13_ = 15.21, *p*<0.001). Significant increases were observed by METH at concentrations of 100 and 1000 µM (*p*<0.001), cocaine at concentrations of 100 and 300 µM (*p*<0.05 and *p*<0.01, respectively) and morphine at a concentration of 100 µM (*p*<0.001), compared with phosphate-buffered saline (PBS)-treated triple slice co-cultures. Single treatment with 3,4-methylenedioxymethamphetamine (MDMA; 0.1–1000 µM) for 30 min tended to increase the extracellular dopamine level, although this increase was not significant (*F*_5,15_ = 1.915, *p* = 0.151).

### Effects of repeated exposure to psychostimulants and morphine on dopamine release

**Figure 3:**
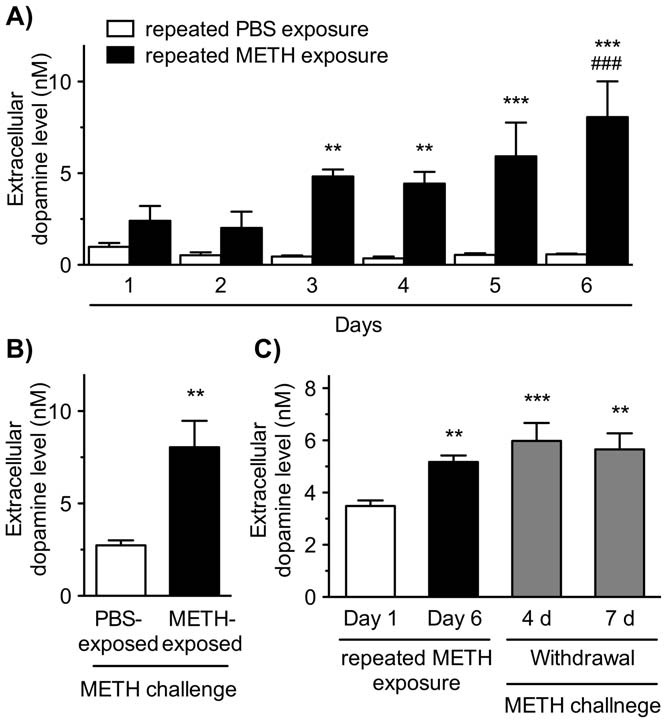
Effect of repeated exposure to METH on dopamine release in the VTA/NAc/mPFC triple slice co-cultures. (A) The triple slice co-cultures were repeatedly exposed to PBS (open column) or METH (10 µM; closed column) in KRH buffer for 30 min every day for 6 days, and the extracellular dopamine level in KRH buffer was determined after each 30 min time point. Values represent the mean of the dopamine concentration ± S.E.M. **p<0.01, ***p<0.001 versus PBS-exposed slice co-cultures. ^###^ *p*<0.001 versus METH-exposed slice co-cultures on the 1st day. *n* = 6. (B) On the day 7, both repeated METH- and PBS-exposed co-cultures were challenged by METH at a concentration of 10 µM in KRH buffer for 30 min, and then the extracellular dopamine level was determined. **p<0.01 versus repeated PBS-exposed slice co-cultures. *n* = 6. (C) The triple slice co-cultures were repeatedly exposed to METH (10 µM) for 6 days, and the extracellular dopamine level was determined at first (Day 1; open column) and 6 days (Day 6; black column). Following the repeated exposure, the slice co-cultures were cultured in normal medium for 4 and 7 days. After the withdrawal periods, the slice co-cultures were challenged with METH (10 µM), and then the extracellular dopamine level was determined (gray column). **p<0.01, ***p<0.001 versus Day 1 (single METH treatment). *n* = 8–17. Values represent means of the dopamine concentration ± S.E.M.

To examine the effect of repeated exposure to METH on dopamine release, the VTA/NAc/mPFC triple slice co-cultures were exposed to METH at a concentration of 10 µM in Krebs-Ringer-Henseleit (KRH) buffer for 30 min every day for 6 days (Fig. 3). The extracellular dopamine level in KRH buffer increased gradually at every METH exposure, while repeated PBS exposure caused no such effect (Fig. 3A). Significant day (*F*_5,25_ = 5.007, *p*<0.01) and drug effects (*F*_1,25_ = 54.72, *p*<0.001) were observed as well as a day×drug significant interaction (*F*_5,25_ = 5.697, *p*<0.01). On day 7, both repeated METH- and PBS-exposed co-cultures were challenged by METH at a concentration of 10 µM for 30 min (Fig. 3B). The METH challenge-evoked dopamine release in the repeated METH-exposed triple slice co-cultures was significantly increased compared with that in the repeated PBS-exposed co-cultures (*p*<0.01). The effect of withdrawal of METH on the repeated METH induced-augmentation of dopamine release was examined (Fig. 3C). When METH was withdrawn for 4 and 7 days following the repeated exposure to METH (10 µM) for 6 days, the METH challenge-evoked dopamine release was still augmented significantly compared with that in the single METH treatment on Day 1 (*p*<0.001 and *p*<0.01, respectively).

**Figure 4:**
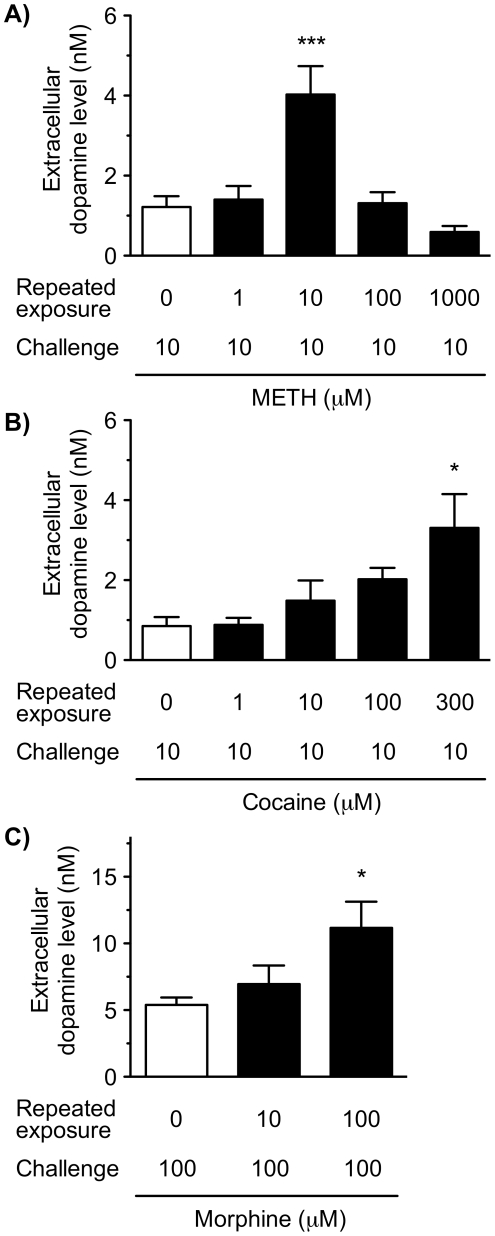
Concentration-dependent effects of repeated exposure to METH, cocaine and morphine on dopamine release in the VTA/NAc/mPFC triple slice co-cultures. The triple slice co-cultures were repeatedly exposed to METH (1–1000 µM; A), cocaine (1–300 µM; B) or morphine (10 and 100 µM; C) in culture medium for 30 min every day for 6 days. On day 7, the slice co-cultures were challenged with METH (10 µM), cocaine (10 µM) or morphine (100 µM), respectively, in KRH buffer for 30 min, and then the extracellular dopamine level was determined. Values represent means of the dopamine concentration ± S.E.M. METH, *n* = 3–6; cocaine, *n* = 3–6; morphine, *n* = 3–7. *p<0.05, ***p<0.001 versus repeated PBS-exposed slice co-cultures.

Next, we examined the concentration-dependence of the repeated METH induced-augmentation of dopamine release (Fig. 4A). The triple slice co-cultures were exposed to PBS or METH (1–1000 µM) in the cultured medium for 30 min every day for 6 days, and then the co-cultures were challenged on day 7 with METH at a concentration of 10 µM in KRH buffer for 30 min. Consistent with the above results, repeated METH exposure significantly increased the extracellular dopamine level following METH challenge (*F*_4,21_ = 8.953, *p*<0.001). Repeated METH exposure displayed a bell-shaped curve of concentration-dependence; the METH challenge-evoked dopamine releases following the repeated METH exposure at concentrations of 100 and 1000 µM were lower than those following the repeated METH exposure at a concentration of 10 µM. Compared with the slice co-cultures repeatedly exposed to PBS, a significant increase in the extracellular dopamine level was observed in the slice co-cultures repeatedly exposed to METH at a concentration of 10 µM (*p*<0.001).

The triple slice co-cultures were exposed to cocaine (1–300 µM) or morphine (10 and 100 µM) for 30 min every day for 6 days, and then the co-cultures were challenged with cocaine (10 µM) or morphine (100 µM), respectively, in KRH buffer for 30 min on day 7 (Fig. 4B and C). Repeated cocaine or morphine exposure significantly increased the extracellular dopamine level (*F*_4,20_ = 3.514, *p*<0.05 and *F*_2,14_ = 4.572, *p*<0.05, respectively) in a concentration-dependent manner. Compared with co-cultures repeatedly exposed to PBS, significant increases in the extracellular dopamine level were observed by the repeated exposure to cocaine at a concentration of 300 µM (*p*<0.05) and morphine at a concentration of 100 µM (*p*<0.05).

### Involvement of NMDA receptors and the mPFC in the repeated METH-induced augmentation of dopamine release

**Figure 5:**
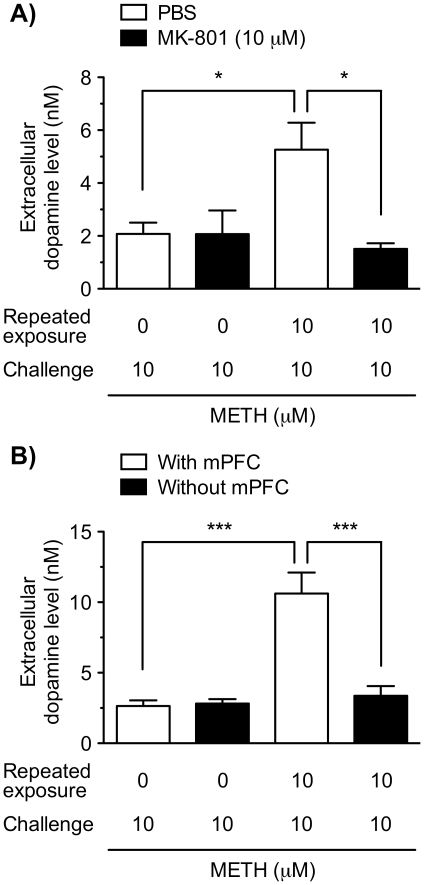
Involvement of NMDA receptors and mPFC in the repeated METH exposure-induced augmentation of dopamine release. (A) The VTA/NAc/mPFC triple slice co-cultures were co-exposed to PBS or METH (10 µM) with (closed column) or without (open column) the NMDA receptor antagonist MK-801 (10 µM) in culture medium for 30 min every day for 6 days. On day 7, the triple slice co-cultures were challenged with METH (10 µM) in KRH buffer for 30 min in the absence of MK-801, and then the extracellular dopamine level was determined. *n* = 3–6. (B) The VTA/NAc/mPFC triple slice co-cultures (open columns) and the VTA/NAc double slice co-cultures without the mPFC slices (closed columns) were repeatedly exposed to PBS or METH (10 µM) in culture medium for 30 min every day for 6 days. On day 7, the triple and double slice co-cultures were challenged with METH (10 µM) in KRH buffer for 30 min, and then the extracellular dopamine level was determined. Values represent means of the dopamine concentration ± S.E.M. *n* = 3–7. *p<0.05, *** *p*<0.001.

To investigate whether NMDA receptors are involved in the induction of the augmentation of dopamine release following the repeated exposure to METH, we examined the effect of the NMDA receptor antagonist MK-801 (Fig. 5A). The VTA/NAc/mPFC triple slice co-cultures were co-exposed to METH (10 µM) with MK-801 (10 µM) or PBS for 30 min every day for 6 days. On day 7, the extracellular dopamine level following the METH challenge (10 µM) was not altered with co-exposure to MK-801 in the slice co-cultures repeatedly exposed to PBS. However, in the slice co-cultures repeatedly exposed to METH, the extracellular dopamine level following METH challenge was significantly lowered by co-exposure to MK-801, compared with co-exposure to PBS (*p*<0.05).

Next, to examine the role of mPFC region in the augmentation of dopamine release following repeated METH exposure, the slice co-cultures composed of the mesencephalic and ventral striatal slices were used without the mPFC slices (Fig. 5B). After repeated exposure to METH (10 µM) for 30 min every day for 6 days, METH challenge (10 µM) on day 7 significantly increased the extracellular dopamine level in the VTA/NAc/mPFC triple slice co-cultures, while the repeated METH-induced augmentation of dopamine release was not observed in the VTA/NAc double slice co-cultures without the mPFC slices. The difference between the slice co-cultures with (triple slice co-cultures) and without (double slice co-cultures) the mPFC slices was significant (*p*<0.001).

## Discussion

To analyze the mechanisms underlying drug sensitization, we reconstructed the mesocorticolimbic system using rat organotypic triple VTA/NAc/mPFC slice co-cultures, as previously reported by Maeda et al. [26]. This system, which retains neural and synaptic function, enables long-term analysis and has clear advantages compared with single cell culture systems. Immunohistochemical analysis revealed that TH-positive dopaminergic cell bodies were observed within the VTA region, and TH-positive neurites extended into the NAc and mPFC, target regions observed *in vivo* [27]. Furthermore, DAT, the direct target of psychostimulants on dopaminergic terminals, was observed in the VTA, NAc and mPFC parts. These results suggest that the triple slice co-culture retained *in vivo* physiology and allowed innervation of the mesocorticolimbic dopaminergic neurons. On the other hand, the mPFC is shown to connect to the NAc through the corticoaccumbens glutamatergic pathway [28]. In the same triple slice co-cultures, Maeda et al. reported that neurites of the mPFC extended into the NAc region, and the corticoaccumbens pathway consisted of functional glutamatergic neurons [26].

In the triple slice co-culture, single treatment with METH and cocaine caused dopamine release in a concentration-dependent manner. METH directly acts on DAT located on dopaminergic nerve terminals and reverses dopamine reuptake, resulting in both reuptake inhibition and release of dopamine. In contrast, cocaine only blocks reuptake of dopamine at dopaminergic nerve terminals [29]. The present findings that cocaine elevated extracellular dopamine levels suggest that the mesocorticolimbic dopaminergic neurons in the triple slice co-culture spontaneously release dopamine, and that the triple slice co-culture has functional DAT and dopaminergic nerve terminals. On the other hand, the effect of MDMA on dopamine release was lower compared with METH and cocaine in the triple slice co-cultures. These results suggest that the direct action of MDMA on the dopaminergic neurons, likely via a DAT-mediated mechanism, is weak, as previously shown [30], [31]. It is known that dopamine release *in vivo* is mediated through indirect mechanisms via MDMA-induced serotonergic activation and direct DAT reversal [30], [31], although the dopaminergic-serotonergic interaction has not been examined in the triple slice co-culture. Similarly, single treatment with morphine caused dopamine release in the triple slice co-cultures that was concentration-dependent. The morphine-evoked dopamine release is considered to be due to inhibition of the inhibitory GABAergic interneurons in the VTA through activation of μ-opioid receptors to activate the mesocorticolimbic dopaminergic neurons [4].

However, the concentrations required for the dopamine release induced by single treatment with METH, cocaine and morphine in the triple slice co-cultures are higher than those reported in other *in vitro* systems, namely cell lines expressing DAT [20], [21] and primary cultures of dopaminergic neurons [22]. In the preparation described here, because the level of dopamine was measured in the absence of monoamine oxidase inhibitors, dopamine might have been rapidly degraded by monoamine oxidases before it spilled over into the KRH buffer. In addition, unlike dissociated cultured cells, the slice culture is thick and its surface is covered by numerous glial cells, which might limit the diffusion of the drugs into the neurons.

A number of *in vivo* studies have indicated that repeated intermittent exposure to psychostimulants and morphine leads to the augmentation of dopamine release from the mesocorticolimbic dopaminergic terminals in the NAc and mPFC, which is thought to be the major cause of the behavioral sensitization ([1], [5]–[10]; but see [13]–[15]). In the present study, we found that repeated exposure of the triple slice co-cultures to METH gradually augmented dopamine release evoked by METH challenge. The augmentation of dopamine release required repeated exposure for several days. Furthermore, the repeated METH-induced augmentation maintained even after the withdrawal of METH for 4 and 7 days, suggesting this phenomenon is based on long-lasting neuroadaptive changes in the dopaminergic neurons. However, the augmented effect of repeated METH exposure was bell-shaped, and the maximum effect was observed at a concentration of 10 µM. It is well known that METH at higher doses induces neurotoxicity, including damage to dopaminergic terminals and neuronal apoptosis [32], [33]. In the triple slice co-cultures, we observed that sustained exposure to higher concentrations of METH (100 and 1000 µM) for 2 days produced marked cytotoxicity, while lower concentrations of METH (1 and 10 µM) exhibited little cytotoxicity in all regions by propidium iodide uptake assay (Figure S1). It has been shown that higher doses of METH to a neurotoxic regimen reduced the dopamine release evoked by potassium or METH in the striatum [34]. Therefore, it is possible that the bell-shaped effect observed in this study may be due to the neurotoxic effect of the higher concentrations of METH. If the depletion of dopamine contents was recovered by the withdrawal of METH for several days, it is possible that the augmentation of dopamine release could be observed or more enhanced.

Similar to METH, the present results show that repeated exposure to cocaine and morphine augmented the dopamine release induced by their challenges. These effects were concentration-dependent. In contrast to METH-induced neurotoxicity, repeated administration of cocaine induced no neurotoxicity for dopaminergic neurons *in vivo* and *in vitro* [35]–[37], and there is no evidence for morphine. Taken together, to our knowledge, this is the first demonstration showing that repeated exposure to psychostimulants and morphine induces augmentation of dopamine release, *i.e.* dopaminergic sensitization *in vitro*.

In addition to the mesocorticolimbic dopaminergic neurons, the glutamatergic neurons have been demonstrated to play a critical role in the induction of behavioral sensitization to psychostimulants and morphine [11], [19], [38]. The present study shows that co-treatment with an NMDA receptor antagonist, MK-801, during repeated METH exposure prevented the repeated METH-induced dopaminergic sensitization, which corresponds to previous *in vivo* findings that the NMDA receptor antagonists prevented the induction of METH-induced behavioral sensitization [18], [39].

The mPFC is a key structure for regulating the firing pattern of mesocorticolimbic dopaminergic neurons [40]. In the present study, the repeated METH-induced dopaminergic sensitization was not observed in the VTA/NAc double slice co-cultures without the mPFC slice, suggesting an essential role of mPFC in the triple slice co-culture. Corresponding to these results, excitotoxic lesion of the mPFC prevented the METH-induced behavioral sensitization *in vivo* [41]. Furthermore, several lines of evidence suggest that the innervations from the mPFC to the NAc and VTA through glutamatergic pathway play an essential role in the induction of behavioral sensitization [11], [41], [42]. Using the same triple slice co-cultures, Maeda et al. showed that a single electrical stimulation of the mPFC slice evoked field excitatory postsynaptic potential (fEPSP) and spontaneous populations spikes in the NAc area of the triple slice co-cultures, which were decreased by NMDA and non-NMDA glutamate receptor antagonists. Furthermore, they found that cocaine attenuated the amplitude of fEPSP in a concentration-dependent manner through activation of D_1_-like, but not D_2_-like, dopamine receptors [26]. These findings suggest that the glutamatergic neurons from the mPFC are linked, at least, to the NAc and are regulated by mesocorticolimbic dopaminergic neurons in the triple slice co-cultures. Thus, it is possible that the corticoaccumbens glutamatergic pathway from the mPFC to the NAc may play a role in the mesocorticolimbic dopaminergic sensitization induced by repeated exposure to METH through activation of NMDA receptors, although we have not determined whether the innervations from the mPFC is linked to the VTA. However, it is also possible that MK-801 blocked the induction of the dopaminergic sensitization directly affecting NMDA receptors in the VTA. Further investigations will be needed to elucidate the roles of glutamatergic pathways from the mPFC to the NAc and VTA.

In summary, we have reconstructed the mesocorticolimbic system *in vitro* using the rat VTA/NAc/mPFC triple slice co-culture and demonstrated repeated dopaminergic sensitization by psychostimulants and morphine. Moreover, our *in vitro* system confirmed that NMDA receptors and the mPFC are essential for the induction of the dopaminergic sensitization, at least, by METH. There are few culture systems that reconstruct the mesocorticolimbic dopaminergic system including the VTA, NAc and mPFC and are also available for long-term analysis *in vitro*. Therefore, this model provides unique opportunities for studying the neural and molecular mechanisms underlying certain processes relevant to behavioral sensitization to drugs of abuse.

## Materials and Methods

### Materials

d,l-METH hydrochloride was purchased from Dainippon Pharmaceutical Co. (Osaka, Japan). Morphine hydrochloride and cocaine hydrochloride were purchased from Takeda Chemical Industries (Osaka, Japan). MDMA hydrochloride was a gift from Dr. Tatsunori Iwamura (Matsuyama University, Matsuyama). (+)-MK-801 hydrogen maleate, an NMDA receptor antagonist, was purchased from Sigma (St Louis, MO, USA). Drugs were dissolved in PBS, and then diluted in the culture medium.

### Preparation of rat mesocorticolimbic triple slice co-cultures

All animal care and experimental procedures were approved by the Kyoto University Animal Research Committee. Organotypic slice co-cultures were prepared according to the methods described previously [26], [43], with slight modifications. Briefly, both male and female Sprague-Dawley rat pups at postnatal days 3–4 (Nihon SLC, Shizuoka, Japan) were anesthetized by hypothermia, and the brains were removed from the skull and separated into two hemispheres. Coronal sections (350 µm thickness) were prepared under sterile conditions with a tissue chopper (Narishige, Tokyo, Japan) at the mesencephalic and telencephalic levels. Tissue samples were dissected for the mesencephalic slice including the VTA, the ventral striatal slice including the NAc and the mPFC slice including the cingulate gyrus and infralimbic cortex, which were identified by visual inspection with the aid of the Atlas of the developing rat brain [44]. Each slice containing the VTA, NAc or mPFC region was arranged so that they contacted each other. Three sets of the triple slices were placed on 30 mm Millicell-CM insert membranes (pre size 0.4 µm; Millipore, Billerica, MA, USA), and the inserts were transfered to a six-well culture plate. Culture medium, consisting of 50% minimal essential medium/HEPES, 25% Hank's balanced salt solution and 25% heat-inactivated horse serum (Gibco, Invitrogen Japan, Tokyo, Japan) supplemented with 6.5 mg/ml glucose and 2 mM l-glutamine, was supplied at a volume of 0.7 ml per well. The triple slice co-cultures were maintained at the liquid/air interface for 18 days in an incubator at 37°C in a 5% CO_2_ humidified atmosphere, and subsequently used in experiments. The culture medium was exchanged for fresh medium on the day following culture preparation, and on every second day thereafter.

### Immunohistochemistry

Slice co-cultures were fixed with 0.1 M phosphate buffer containing 4% paraformaldehyde and 4% sucrose for 2 hr. After washing with PBS, fixed cultures were permeabilized and blocked with PBS containing 0.2% Triton X-100 and 10% fetal calf serum for 1 hr. The cultures were then incubated overnight at 4°C with rabbit anti-TH polyclonal antibody (1∶200, AB-152, Chemicon International, Temecula, CA, USA), and washed with PBS three times. The cultures were incubated for 1 hr in a secondary antibody solution containing either Alexa Fluor 488 goat anti-rabbit IgG (1∶200, Molecular Probes, Eugene, OR, USA), or biotinylated anti-rabbit IgG (1∶200, Vector Laboratories, Burlingame, CA, USA) and washed with PBS three times.

For immunofluorescent staining, the cultures were transferred to glass slides and cover-slipped using Vectashield hard-set mounting medium (Vector Lab). Immunofluorescence was visualized using a Nikon Diaphot 200 microscope equipped with a laser scanning confocal imaging system (MRC-1024, Bio-Rad Laboratories, Hercules, CA). For immunostaining using the biotin-avidin peroxidase method, cultures were treated for 1 hr with avidin–biotinylated horseradish peroxidase complex (Vectastain Elite ABC kit, Vector Lab) at room temperature. After a further wash with 50 mM Tris-buffered saline, peroxidase was visualized with 0.07% 3,3′-diaminobenzidine and 0.018% H_2_O_2_. Specimens were dehydrated through a graded ethanol series and mounted on glass slides with glycerol for observation with a microscope using bright-field illumination.

### Western blot

After 18 days culture, slice co-cultures were separated into VTA, NAc and mPFC parts under dissecting microscope, and these parts from 4 culture inserts were harvested in 100 µL Lysis buffer (20 mM Tris-HCl, 1% Triton-X (Nacalai tesque, Kyoto, Japan) and 1% protease inhibitor cocktail (Merck, Darmstadt, Germany)). After harvest, these parts were homogenized, sonicated and centrifuged at 600 g for 5 min, and protein concentration of resulting supernatants were measured by the bicinchoninic acid protein assay (Thermo Fisher, MA, USA). Equal volume of 2× Laemmli sample buffer were added to supernatants and stored at −20°C until use. Thawed samples were analyzed by SDS-PAGE, transferred to polyvinylidene difluoride membrane and probed with anti-dopamine transporter antibody (1∶1000; D6944, Sigma-Aldrich, St Louis, MO, USA) or anti-Na^+^/K^+^-ATPase (1∶10000; Abcam, Cambridge, UK), followed by probing with horseradish peroxidase-conjugate secondary antibody. Proteins were visualized by Immobilon Western HRP substrate (Millipore, Billerica, MA, USA) and detected by X-ray film.

### Measurement of dopamine release

Measurement of extracellular dopamine levels was performed as previously described [43], with slight modifications. Briefly, for measurement of extracellular dopamine levels, culture inserts were transferred and washed in 0.7 mL KRH buffer (146 mM NaCl, 2.7 mM KCl, 1 mM MgCl_2_, 1.2 mM CaCl_2_, 10 mM D-glucose, 15 mM HEPES, 5 mM HEPES-Na, 0.2 mM ascorbic acid; pH 7.4) three times. After the washes, the inserts were preincubated in KRH buffer for 15 min, then transferred to 0.7 mL KRH buffer containing drugs and incubated for 30 min. After incubation, KRH buffer was collected into vials, and acid solution was added that contained 1 M HClO_4_, 100 mM Na_2_S_2_O_5_ and 10 mM EDTA. To quantify dopamine concentration, the collected aliquot (20 µl) was automatically injected and analyzed by high performance liquid chromatography with an electrochemical detection system (Eicom, Kyoto, Japan). Dopamine was quantified by reference to a linear calibration curve ranging from 0.1 to 100 nM. The detection limits for dopamine were estimated to be approximately 10 pM per 20 µl sample.

### Statistical analysis

Data are presented as the mean ± S.E.M. Differences between two groups were compared using the Student's *t*-test. Data with more than two groups were compared by one-way analysis of variance (ANOVA), followed by the Dunnett's multiple comparison test. The time-course data was analyzed by two-way ANOVA for repeated measures, followed by the Bonferroni *post-hoc* test. Differences of *p*<0.05 were considered statistically significant.

## Supporting Information

Figure S1 **Effect of sustained exposure to METH on the propidium iodide (PI) uptake in the VTA/NAc/mPFC triple slice co-cultures.** The triple slice co-cultures were exposed to PBS, METH (1–1000 µM) or NMDA (300 µM) in the presence of PI (5 µg/ml). After 2 days incubation, the PI fluorescence of each slice was observed with an inverted fluorescence microscope with a rhodamine filter set. The triple slice co-cultures exposed to *N*-methyl-d-aspartate (NMDA; 300 µM) for 2 days were used to determine the degree of the standard injury. Representative photomicrographs of PI fluorescence in the NAc (upper), mPFC (middle) and VTA (bottom) regions, respectively, are shown. Sustained exposure of the slice co-cultures to METH for 2 days exhibited concentration-dependent increases in PI fluorescence in each of the NAc, mPFC, and VTA regions. In all regions, a small number of PI positive cells was observed with 10 µM METH, and marked increases of PI positive cells were observed with 100 and 1000 µM METH. The total number of PI positive cells in the mPFC region was greater than in the other two regions. This is likely due to the fact that in the mPFC region, there were a greater total number of cells in the original triple slice co-cultures.
(TIF)
