# Supplementary figures and images for: Repeated Exposure to Methamphetamine, Cocaine or Morphine Induces Augmentation of Dopamine Release in Rat Mesocorticolimbic Slice Co-Cultures
Source: PLoS One. 2011 Sep 30;6(9):e24865. doi: 10.1371/journal.pone.0024865 (PMC3184101; doi:10.1371/journal.pone.0024865)

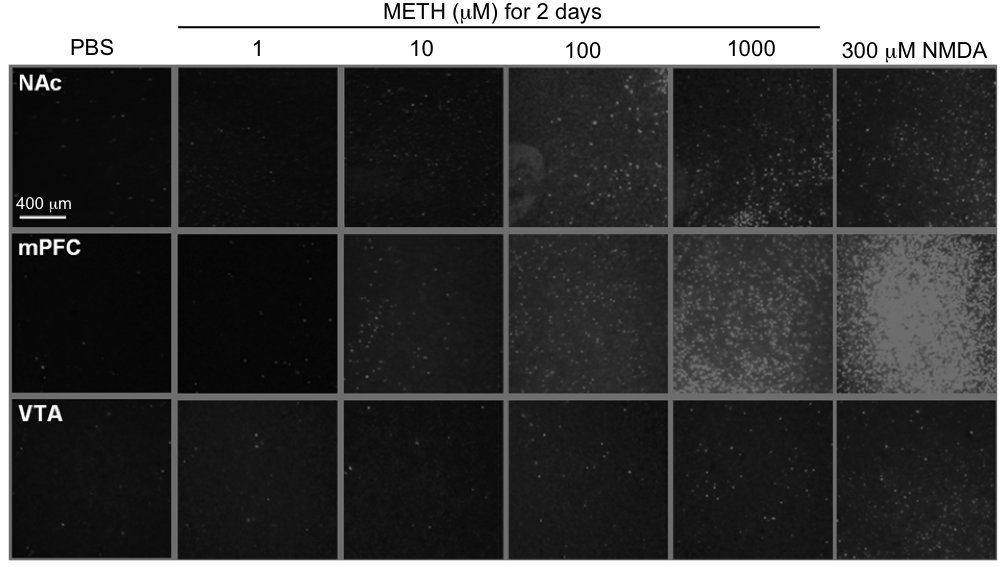

Supplement: Figure S1 — Effect of sustained exposure to METH on the propidium iodide (PI) uptake in the VTA/NAc/mPFC triple slice co-cultures. The triple slice co-cultures were exposed to PBS, METH (1–1000 µM) or NMDA (300 µM) in the presence of PI (5 µg/ml). After 2 days incubation, the PI fluorescence of each slice was observed with an inverted fluorescence microscope with a rhodamine filter set. The triple slice co-cultures exposed to N-methyl-d-aspartate (NMDA; 300 µM) for 2 days were used to determine the degree of the standard injury. Representative photomicrographs of PI fluorescence in the NAc (upper), mPFC (middle) and VTA (bottom) regions, respectively, are shown. Sustained exposure of the slice co-cultures to METH for 2 days exhibited concentration-dependent increases in PI fluorescence in each of the NAc, mPFC, and VTA regions. In all regions, a small number of PI positive cells was observed with 10 µM METH, and marked increases of PI positive cells were observed with 100 and 1000 µM METH. The total number of PI positive cells in the mPFC region was greater than in the other two regions. This is likely due to the fact that in the mPFC region, there were a greater total number of cells in the original triple slice co-cultures. (TIF) [file pone.0024865.s001.tif]
